# The Effect of Calcium and Halide Ions on the Gramicidin A Molecular State and Antimicrobial Activity

**DOI:** 10.3390/ijms21176177

**Published:** 2020-08-27

**Authors:** Kathleen D. Carillo, Chi-Jen Lo, Der-Lii M. Tzou, Yi-Hung Lin, Shang-Ting Fang, Shu-Hsiang Huang, Yi-Cheng Chen

**Affiliations:** 1 Taiwan International Graduate Program, Sustainable Chemical Science and Technology (SCST), Academia Sinica, Nankang, Taipei 11529, Taiwan; kjcarillo@yahoo.com; 2 The Department of Applied Chemistry, National Chiao-Tung University, Hsinchu 30013, Taiwan; 3 Institute of Chemistry, Academia Sinica, Nankang, Taipei 11529, Taiwan; tzougate@gate.sinica.edu.tw; 4 Metabolomics Core Laboratory, Healthy Aging Research Center, Chang Gung University, Taoyuan 333, Taiwan; chijenlo@gmail.com; 5 Department of Applied Chemistry, National Chia-Yi University, Chia-Yi 60004, Taiwan; 6 Experimental Facility Division, Industrial Application Group, National Synchrotron Radiation Research Center, Hsinchu Science Park, Hsinchu 30076, Taiwan; yihung@nsrrc.org.tw; 7 Department of Medicine, MacKay Medical College, New Taipei City 252, Taiwan; farmting@hotmail.com (S.-T.F.); sshuang33@mmc.edu.tw (S.-H.H.)

**Keywords:** gramicidin A, halide, molecular state, antimicrobial activity, dissociation

## Abstract

Gramicidin A (gA) forms several convertible conformations in different environments. In this study, we investigated the effect of calcium halides on the molecular state and antimicrobial activity of gramicidin A. The molecular state of gramicidin A is highly affected by the concentration of calcium salt and the type of halide anion. Gramicidin A can exist in two states that can be characterized by circular dichroism (CD), mass, nuclear magnetic resonance (NMR) and fluorescence spectroscopy. In State 1, the main molecular state of gramicidin A is as a dimer, and the addition of calcium salt can convert a mixture of four species into a single species, which is possibly a left-handed parallel double helix. In State 2, the addition of calcium halides drives gramicidin A dissociation and denaturation from a structured dimer into a rapid equilibrium of structured/unstructured monomer. We found that the abilities of dissociation and denaturation were highly dependent on the type of halide anion. The dissociation ability of calcium halides may play a vital role in the antimicrobial activity, as the structured monomeric form had the highest antimicrobial activity. Herein, our study demonstrated that the molecular state was correlated with the antimicrobial activity.

## 1. Introduction

Gramicidin is an antimicrobial peptide that is a byproduct of *Bacillus brevis* during sporulation [1]. Gramicidin contains alternating L- and D-amino acids in its primary sequence and has three isoforms—gramicidin A (gA), gramicidin B (gB) and gramicidin C (gC)—in which residue 11 is Trp for gA, Phe for gB and Tyr for gC [2,3]. Gramicidin adopts a number of different conformations in different environmental conditions [4,5,6,7]. In organic solvents such as methanol, gramicidin forms a β-sheet-like double-strand helical structure, in which two monomers are interwound similarly as DNA double helices [4,6,8]. This double-strand helical dimer has four distinct conformations that can be either parallel or antiparallel, left- or right-handed double-stranded helices [6,7,8,9]. These double-helical conformers are interconvertible and have distinct circular dichroism (CD) spectra [6,8]. On the other hand, in polar solvents such as trifluoroethanol (TFE), gramicidin forms a single-stranded and right-handed helical monomer [4,10,11,12]. Two of the helical monomers can form a head-to-head channel dimer in a lipid environment [4,11,12]. This channel can specifically translocate small monovalent cations such as H^+^, Na^+^ and K^+^ across membranes [4,12,13,14].

Gramicidin’s double-stranded helical dimer can interact with monovalent or divalent cations [15,16,17,18,19,20,21]. The complex of gramicidin A/monovalent cation exists in a left-handed antiparallel double-helical conformation in methanol [15,16] and has a distinct CD spectrum compared to that of the ion-free form [17]. The binding mechanism is a cooperative mode, and the binding affinities are roughly related to the size of the monovalent cations. The order of the binding affinities is Cs > Rb >> K > Li [17]. Previous studies demonstrated that divalent cations could cause the gramicidin A mixture in methanol to form one specific conformation [18,19]. The CD spectrum of the gramicidin A/divalent cation complex is very different from that of the gramicidin A/monovalent cation complex in methanol [19]. The structure of the gramicidin A/Ca complex determined by the solution NMR technique appears to be a left-handed parallel double helical conformation with Ca bound at the N-terminal mouth of the double helical dimer [20].

Conversion between conformers has been studied in lipid environments [21,22,23,24,25]. In membrane lipid environments, the rate of conversion from the double-stranded helical dimer into the channel dimer is dependent on environmental factors, such as temperature and ionic strength [21,22,23]. Both CD and fluorescence spectroscopies have been used to examine the rates of conversion of the double-stranded helical dimer to the single-stranded helical dimer in synthetic lipid membranes [21,23]. However, the conversion between the double-strand helical conformers in organic solvents and different ions has not been studied.

Gramicidin A is a short peptide antibiotic effective against bacteria and fungi [26]. The antimicrobial activity of gramicidin A has been associated with the disruption of membrane lipids [27]. We have previously demonstrated that the antimicrobial activity of gramicidin A may also be associated with the formation of free radicals via the disruption of NADH/NAD^+^ synthesis [28]. Recently, the antimicrobial function of gramicidin A was also linked with the formation of ion channels [29]. However, the relationship between antimicrobial activity and molecular state is not entirely clear.

In the present study, we revisited and characterized the conformational and molecular states of gA in the presence of different calcium and halide ions using circular dichroism (CD), nuclear magnetic resonance (NMR) and state-of-art mass spectroscopy, and found that gramicidin A exists in two molecular states in the presence of calcium halides. In State 1, the main effect of calcium halides on gramicidin A is to convert the conformers into one single conformation. In State 2, calcium halides can induce a dissociation of the gramicidin A dimer into monomers and the denaturation of the gramicidin A monomer. A new finding revealed that the different halide anions may play a role in the molecular state and conformation of gramicidin A. The molecular state and conformation induced by calcium halide can further influence the antimicrobial activity of gramicidin A.

## 2. Results

### 2.1. Circular Dichroism Spectroscopy

Gramicidin A adopts several conformers in methanol [4,6,7,8]. We characterized the conformational and molecular states of gramicidin A in the presence of calcium halides and in methanol using several spectroscopic techniques including CD, NMR and mass spectroscopy.

**Figure 1:**
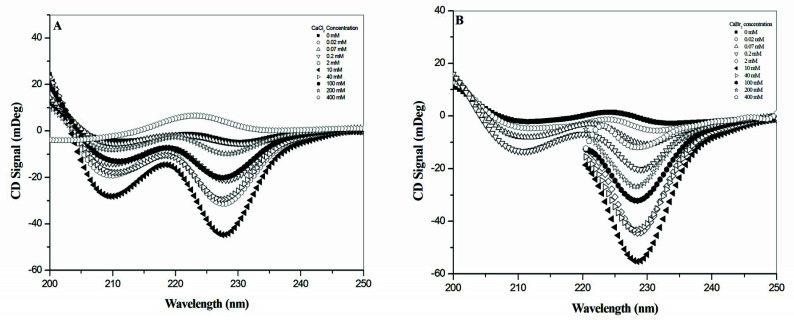
Circular dichroism (CD) spectra of gramicidin A in the presence of CaCl_2_ and CaBr_2_. One hundred micromolar gramicidin A was titrated with either (**A**) CaCl_2_ or (**B**) CaBr_2_ in methanol. All CD spectra were recorded from 200 to 250 nm.

Figure 1A,B shows the CD spectra for 100 μM gramicidin A titrated with CaCl_2_ and CaBr_2_. For CaBr_2_, the CD spectra below 220 nm became noisy at the concentration ≥ 0.2 mM due to the strong adsorption from the Br^−^ anion. Therefore, we were unable to collect CD spectra from 200 to 220 nm. A similar result was also obtained when we titrated gramicidin A with CaI_2_. An even stronger absorption arising from the I^−^ anion prevented us from collecting any reasonable CD spectra. Hence, we were unable to study the effect of CaI_2_ on the gramicidin A conformation using CD spectroscopy.

**Figure 2:**
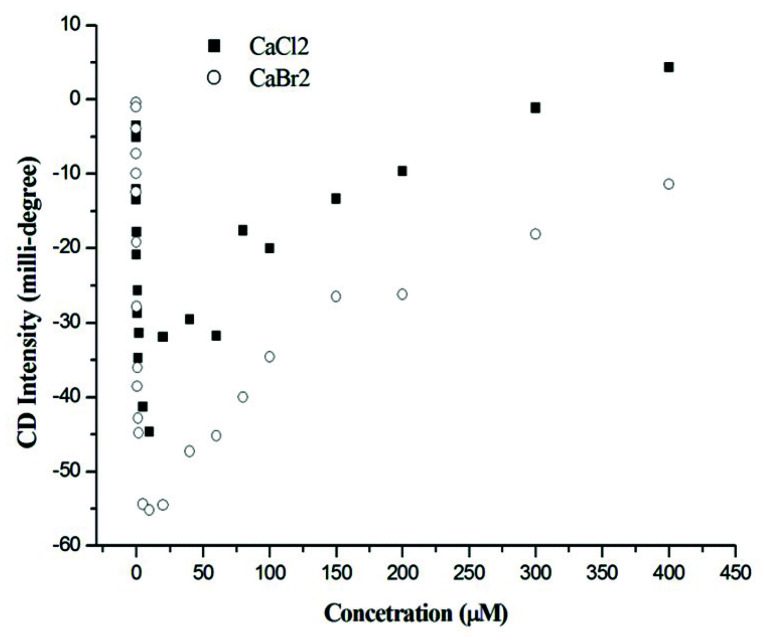
The intensity of the CD signal at 228 nm vs. the Ca salt concentration. The titration curves for one hundred micromolar gramicidin A titrated with CaCl_2_ (⯀) and CaBr_2_ (○) in methanol, respectively.

At salt concentrations ≤ 10 mM, the CD spectra showed similar spectral patterns, with the two negative peaks located at 208 and 228 nm for both CaCl_2_ and CaBr_2_. The intensity of the two negative peaks increases with an increase in the salt concentration and reaches its maximum at around 10 mM. At salt concentrations > 10 mM, these two negative peaks gradually converted into a single broadened positive peak located at ~225 nm. The change in intensity was different between the Cl^−^ and Br^−^ anions. For CaCl_2_, the two negative peaks converted into a positive peak at a salt concentration higher than 300 mM, whereas the two negative peaks did not convert into a positive peak at 400 mM for CaBr_2_. Figure 2 shows the titration curve of the intensity at 228 nm vs. salt concentration. According to the titration curves (Figure 2), gramicidin A may exist in two states for both CaCl_2_ and CaBr_2_. The CD results suggest that gramicidin A may adopt different conformations in these two states for CaCl_2_ and CaBr_2_.

### 2.2. Nuclear Magnetic Resonance Spectroscopy

**Figure 3:**
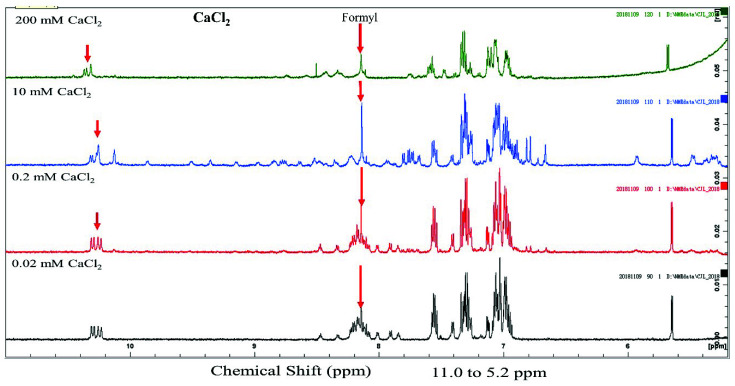
Nuclear magnetic resonance (NMR) spectra of gramicidin A in the presence of CaCl_2_. From bottom to top, the NH region, 5.0–10.5 ppm, is shown for 100 μM gramicidin A with 0.02, 0.2, 10 and 200 mM CaCl_2_ in 90% d4-methanol/10% methanol.

**Figure 4:**
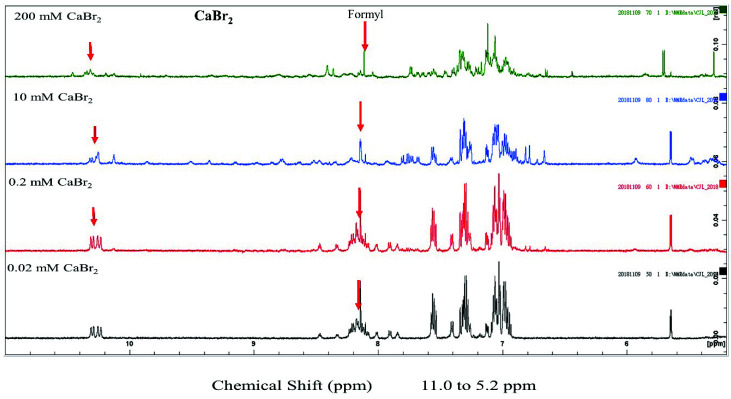
NMR spectra of gramicidin A in the presence of CaBr_2_. From bottom to top, the NH region, 5.0–10.5 ppm, is shown for 100 μM gramicidin A with 0.02, 0.2, 10 and 200 mM CaBr_2_ in 90% d4-methanol/10% methanol.

The conformation of gramicidin A in the presence of calcium halide salts was examined using 1D proton NMR spectroscopy. The 1D proton NMR spectra of gramicidin A in the presence of CaCl_2_, CaBr_2_ and CaI_2_ are shown in Figure 3 and Figure 4, and Figure S1 in Supplementary Information, respectively. Two spectral features in the NMR spectrum are particularly interesting. The chemical shifts at 8.0–8.2 ppm were assigned as the N-terminal formyl group [19,30]. The resonance assignment for the chemical shifts at 10.0–10.5 ppm was not determined in this study and required further 2D experiments.

At a low salt concentration, i.e., 0.2 mM, the proton signal of the formyl group was multiple resonance for CaCl_2_, CaBr_2_ and CaI_2_, indicating that a mixture of conformers is present under this condition. Unlike the resonance pattern of the formyl group at a concentration of 0.2 mM, the resonance of the formyl proton became a single peak at a concentration of 10 mM for CaCl_2_, CaBr_2_ and CaI_2_, indicating that a single conformation was present. The dispersion of these NMR spectra was well resolved at 5.0~10.0 ppm compared that of to the spectra obtained at a concentration of 0.2 mM. The well-resolved peaks at 10–10.5 ppm were not well separated compared to those at 0.2 mM. According to a previous study [20], gramicidin A may form a left-handed parallel double helix at 10 mM calcium salt.

At a high salt concentration, i.e., 200 mM, the NMR spectra of gramicidin A were very different compared to those obtained at 10 mM or 0.2 mM and dependent on the different types of halide anion as well. At a concentration of 200 mM CaCl_2_, the individual peaks in the NMR spectrum were less dispersed compared to those in the NMR spectrum of gramicidin A at 10 mM CaCl_2_. A previous study has hypothesized that gramicidin A may possibly exist in a mixture of unstructured and structured monomers under this condition [19]. The NMR spectrum for CaI_2_ was even more separated, and peaks for a specific type of amino acid even coincided, indicating that gramicidin A may be further denatured rather than forming a structured/unstructured monomer in CaBr_2_ and CaI_2_. Clearly, the types of halide anions have different impacts on the conformation of gramicidin A at high salt concentrations.

### 2.3. Mass Spectroscopy

**Figure 5:**
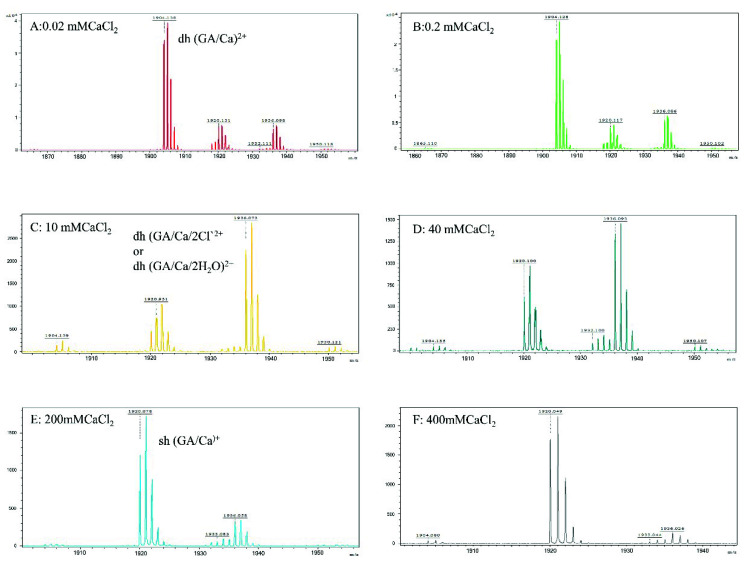
Mass spectra of gramicidin A in the presence of CaCl_2_. The samples containing 100 μM gramicidin A with CaCl_2_ at 0.02 (**A**), 0.2 (**B**), 10 (**C**), 40 (**D**), 100 (**E**) and 400 mM (**F**) in methanol were mixed with the matrix for mass analyses.

**Figure 6:**
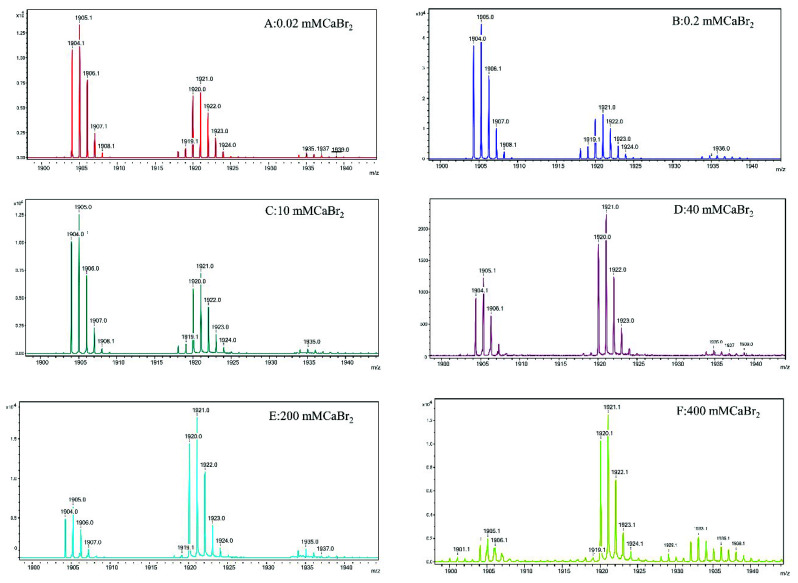
Mass spectra of gramicidin A in the presence of CaBr_2_. The samples containing 100 μM gramicidin A with CaBr_2_ at 0.02 (**A**), 0.2 (**B**), 10 (**C**), 40 (**D**), 100 (**E**) and 400 mM (**F**) in methanol were mixed with the matrix for mass analyses.

To further characterize the molecular state of gramicidin A at the different concentrations of calcium salt and with the different types of halide anion, we applied MALDI-TOF mass spectrometry to study the molecular state of gramicidin A. Figure 5 and Figure 6 show the mass spectra of gramicidin A with CaCl_2_ and CaBr_2_ at various concentrations, respectively. The mass spectra of gramicidin A treated with CaI_2_ are delineated in Figure S2.

The peaks at *m*/*z* 1902–1905 were assigned as (gA dimer/Ca)^2+^, while the peaks at *m*/*z* 1920–1921 were assigned as (gA monomer/Ca/H)^+^. In the case of gA/CaCl_2_, a new peak at *m*/*z* 1936 was assigned as (gA dimer/Ca+2Cl)^2+^ or (gA dimer/Ca+4H_2_O)^2+^, while this peak was not observed in the cases of CaBr_2_ (Figure 6) and CaI_2_ (Figure S2). Due to the high oxidative strength of iodine, we were unable to obtain a reasonable mass for gramicidin A/CaI_2_ at concentrations higher than 100 mM (Figure S2).

The molar ratios of the dimer/monomer of gramicidin A in the presence of different salt concentrations and halides were calculated using the intensity of 1905(+1936 for CaCl_2_)/1920. The molar ratios of the dimer/monomer are summarized in Table 1. For CaCl_2_ and CaBr_2_, the majority of the gramicidin A at a low salt concentration, i.e., 0.2 mM, was in the dimeric state. The molar ratios of the dimer/monomer were 6.0 and 2.0 for CaCl_2_ and CaBr_2_, respectively. At medium salt concentrations, i.e., 10 and 40 mM, the molar ratios of the dimer/monomer for gA in the presence of CaCl_2_ were two- to four-fold higher than those for gA in the presence of CaBr_2_. At a higher salt concentration, i.e., 100 mM, gramicidin A was in the monomeric state for both CaCl_2_ and CaBr_2_. In general, CaBr_2_ induced the formation of the monomer more efficiently than CaCl_2_ did. This effect was even more profound for CaI_2_, as most gramicidin A species were monomeric, even at very low CaI_2_ concentrations. The value of the dimer/monomer ratio might be underestimated. As a α-cyano-4-OH cinnamic acid (CHCA) matrix dissolved in 50% acetonitrile/50% distilled H_2_O was mixed with the sample in methanol, the acetonitrile/water solvent system favored monomer formation. This may shift the equilibrium toward the monomer and underestimate the dimer/monomer ratio. However, this factor does not affect the conclusion.

### 2.4. Tryptophan Fluorescence Spectroscopy

**Figure 7:**
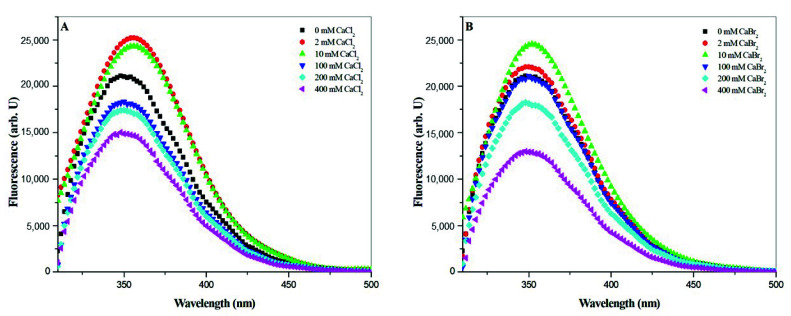
Steady-state fluorescence spectra of gramicidin A in the presence of (**A**) CaCl_2_ and (**B**) CaBr_2_. One hundred μM of gramicidin A titrated with CaCl_2_ and CaBr_2_ in methanol. In the fluorescence spectra, the concentration of CaCl_2_ or CaBr2 increases from top to bottom.

Gramicidin A contains four tryptophan residues at its C-terminus. The Trp residues are a good indicator for examining the influence of the microenvironment on gramicidin A’s molecular state. The steady-state tryptophan fluorescence spectra of gramicidin A titrated with CaCl_2_ and CaBr_2_ are shown in Figure 7A,B, respectively. For both CaCl_2_ and CaBr_2_, there is a fluorescent peak located at around 350 nm. At calcium salt concentrations ≤ 10 mM, the fluorescence intensity increased with an increase in the calcium salt concentration, whereas at calcium salt concentration > 10 mM, the intensity gradually decreased with an increase in the salt concentration. The peak wavelength also underwent a redshift with an increase in salt concentration at the salt concentrations ≤ 10 mM and a blueshift with an increase in salt concentration at the salt concentrations > 10 mM, indicating that calcium halide salts may have an impact not only on the gramicidin A backbone but also on the indole rings of the four Trp residues. The blueshift of the wavelength and decrease in intensity at high concentrations of Ca salts, particularly CaBr_2_ and CaI_2_, may suggest that gramicidin A undergoes denaturation [31,32]. This reinforces the results obtained in the NMR studies.

### 2.5. Antimicrobial Activities

**Figure 8:**
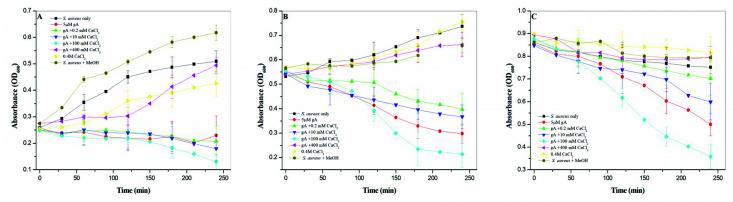
The antimicrobial activity of gramicidin A in the presence of CaCl_2_ (**A**–**C**). In the antimicrobial activity assay, stock solutions were prepared containing 100 μM gramicidin A with 0, 0.2, 10, 100 and 400 mM CaCl_2_ in methanol. The stock solutions were then diluted into *S. aureus* culture with a final solution containing 5 μM gramicidin A. (**A**), lag phase; (**B**), exponential phase; and (**C**), stationary phase.

Our spectroscopic studies demonstrate that the backbone conformation and side-chain microenvironment of gramicidin A were profoundly affected by the concentration of calcium and the types of halide anion. We then examined the antimicrobial activity of gramicidin A in the presence of CaCl_2_. Figure 8 shows the antimicrobial activity of gramicidin A with different concentrations of CaCl_2_ against *Staphylococcus aureus* at different bacterial growth phases. The antimicrobial activity of gramicidin A was inhibited in the pretreatment with 400 mM CaCl_2_ compared to the control (without gA) at all growth phases. In the lag phase, the growth of *S. aureus* was significantly inhibited by gramicidin A with or without CaCl_2_. In the exponential phase, the antimicrobial activity of gramicidin A was significantly inhibited in the pretreatment with 100 mM CaCl_2_ or in the absence of CaCl_2_ compared to that with the pretreatment of 0.2 and 10 mM CaCl_2_. In the late-exponential-to-stationary phase, the antimicrobial activity of gramicidin A in the pretreatment with 100 mM CaCl_2_ was more effective than that in the pretreatments with other CaCl_2_ concentrations. The antimicrobial activity of gramicidin A was similar in CaBr_2_. The most effective concentration regarding antimicrobial activity for CaBr_2_ was 10 mM rather than 100 mM (Figure S3).

## 3. Discussion

The effect of calcium cations on gramicidin A’s conformation was previously studied using infrared (IR) and CD spectroscopic techniques [18,19,20,33]. However, the detailed molecular and conformational states of gramicidin A under the effects of calcium cations and halide anions required further verification. In the present study, we applied state-of-the-art mass, NMR and fluorescence spectroscopy to clarify the detailed molecular and structural states of gramicidin A in the presence of calcium cations and halide anions. We used the obtained structural and molecular information to examine the effect of calcium halides on the antimicrobial activity of gramicidin A.

Our results obtained from CD and NMR spectroscopies are similar to those from the studies in CaCl_2_ by Wallace and colleagues [18,19]. According to the combination of CD and NMR spectra, the effect of calcium halides on the conformation of gramicidin A reached a turning point at the concentration of 10 mM for CaCl_2_, CaBr_2_ and CaI_2_, indicating that gramicidin A may exist in two molecular states, State 1 at salt concentrations ≤ 10 mM and State 2 at salt concentration > 10 mM. The observations obtained with CD and NMR are consistent with the fluorescence spectroscopic studies. In the fluorescence spectra, the intensity increased with an increase in the salt concentration, demonstrated a redshift in wavelength at concentrations ≤ 10 mM, and decreased with an increase in concentration with a blueshift in wavelength at concentrations > 10 mM. These results reinforce that there are two states for gramicidin A upon the titration of calcium halides.

From the CD and NMR spectra, we demonstrated that the main effect of calcium salt is to drive a mixture of conformers into a single species in State 1. This is consistent with the previous study by Chen and Wallace [19]. In addition, we further found that this effect on gramicidin A conformation was dependent on the calcium concentration and independent of the types of halide anions. The progress of the NMR spectral change vs. concentration was similar for all different halide anions, indicating that the conformational change of gramicidin A is only induced by Ca^2+^ cations. The 1D NMR spectra of gramicidin A at 10 mM showed a similar resonance pattern at the amide proton region for CaCl_2_, CaBr_2_ and CaI_2_. These NMR spectra are similar to those in a previous NMR study [20], suggesting that the conformation of gramicidin A may form a left-handed parallel double helix under this condition.

The most interesting findings in the present study are from the mass spectroscopy analyses. The analyses of the mass spectra for all calcium halides suggest that the molecular state of gramicidin A is a mixture of the dimer and monomer in State 1. The major form of gramicidin A exists in a dimeric form for both CaCl_2_ and CaBr_2_ but not for CaI_2_. The dimer content decreased with an increase in the salt concentration, indicating that the addition of the calcium salt drove a dissociation of gramicidin A dimers into monomers. The dissociation of the gramicidin A dimer was more effective with CaI_2_ or CaBr_2_ than with CaCl_2_, suggesting that the dissociation is highly dependent on the type of halide anion. The order by dissociation ability is I^−^ > Br^−^ > Cl^−^.

The reason for this phenomenon is not apparent. A possible reason may be the ionic radii of the halide anion. A peak at *m*/*z* 1936 assigned as (gA dimer/Ca^+^+2Cl)^2+^ only appeared in the mass spectrum of CaCl_2_ but not in the cases of CaBr_2_ and CaI_2_. A similar observation was reported by Zhou and colleagues [34]. These results suggest that the gramicidin A dimer may interact only with Cl^−^, possibly inside the pore, but not with Br^−^ or I^−^. As the ionic radii of Br^−^ and I^−^ are larger than that of Cl^−^, both the Br^−^ and I^−^ anions are too large to accommodate them inside the dimer pore of gramicidin A. The interaction of Cl^−^ ions with gramicidin A may stabilize the dimer and reduce the dimer-to-monomer conversion rate. Therefore, the molar ratio of dimer/monomer in CaCl_2_ is much higher than the molar ratios in CaBr_2_ and CaI_2_. However, the exact reason needs to be further verified with a detailed structure.

In State 2, our CD spectra are also similar to those in a study by Chen and Wallace [19]. The two negative CD peaks gradually converted into a single positive peak in CaCl_2_, indicating that gramicidin A may adopt a different conformation. Chen and Wallace proposed that gramicidin A may be a mixture of unstructured and structured monomers in this condition. Analyses of the mass spectra indicate that the molar ratios of dimer/monomer were all less than 1 for most calcium halides except for 40 mM CaCl_2_. Our results support the augment that the molecular state of gramicidin A should be a monomer in State 2. Similarly, the formation of monomers is also anion-type dependent.

We examined the structural state of gramicidin A using NMR spectroscopy. The results showed that the effect of halide anions on the gramicidin A’s conformation was very different. Gramicidin A is a mixture of unstructured and structured monomers in the presence of 200 mM CaCl_2_. This is consistent with a previous study by Chen and Wallace [19]. The conformation of gramicidin A in 200 mM CaBr_2_ was more unstructured compared to that in 200 mM CaCl_2_. The unstructured effect was even more profound with the addition of 200 mM CaI_2_, indicating that Br^−^ and I^−^ anions may induce denaturation in gramicidin A. The denaturing effect of halide anions on gramicidin A is well correlated with the trend of the dimer-to-monomer dissociation of gramicidin A: I^−^ > Br^−^ > Cl^−^.

The denaturation of gramicidin A induced by halide was further confirmed by the fluorescence spectroscopy. During protein unfolding, the fluorescence spectrum usually undergoes a blueshift in the maximum wavelength and a decrease in the intensity [31,32,35]. In State 2, the fluorescence intensity decreased with an increase in the salt concentration, and the maximum wavelength underwent a blueshift with an increase in the salt concentration., suggesting that the gramicidin A monomer may be denatured by halide anions. Taken together, these results suggest that the halide anions, particularly Br^−^ and I^−^, may play a role in the denaturation of gramicidin A. The denaturation ability of halide anions, particularly Br^−^ and I^−^, could be taken to account for the nature of the dissociation of gramicidin A.

The antimicrobial activity of gramicidin A increased with an increase in the calcium concentration but not for 400 mM CaCl_2_. At 400 mM CaCl_2_, the antimicrobial activity of gramicidin A was significantly inhibited. The antimicrobial activity of gramicidin A is in the order 100 mM CaCl_2_ >> 0 mM CaCl_2_ > 10 mM CaCl_2_ ≈ 0.2 mM CaCl_2_ >> 400 mM CaCl_2_. The molecular states of gramicidin A/CaCl_2_ complexes in methanol may be changed when they are added to culture medium, as the addition of the gramicidin A in methanol into culture medium may drive the conversion of dimers into monomers. Therefore, the antimicrobial activity may have been overestimated in the present study. However, this does not affect the present conclusion.

The results obtained for the antimicrobial activity vs. CaCl_2_ concentration suggest that the monomeric gramicidin A had the most effective inhibitory ability against bacterial growth. A previous study by Jadhay et al. suggested that gramicidin A in channel form mediated the most effective antimicrobial activity against Gram-positive bacteria [29]. The formation of gramicidin channels in membranes is highly dependent on the environment [36,37]. In 100 mM CaCl_2_, the main form of gramicidin A is monomeric. Gramicidin A in the presence of 100 mM CaCl_2_ can readily form ion channels in bacterial cell walls and, hence, shows the most effective antimicrobial activity. On the other hand, the major form of gramicidin A is dimeric in 0, 0.2 and 10 mM CaCl_2_. When bacteria are treated with gramicidin A in these conditions, it takes time to convert the dimers into monomers. Therefore, gramicidin A in these conditions was less effective in terms of antimicrobial activity when compared to the case at 100 mM CaCl_2_.

The case of gramicidin A in 400 mM CaCl_2_ was different from that at 100 mM CaCl_2_. Gramicidin A may exist largely in the denatured state in 400 mM CaCl_2_. This may cause the loss of the antimicrobial function. However, we could not exclude the possibility that the decrease in antimicrobial activity may have been due to the blocking of the gramicidin A channel by the high concentration of calcium cations, as calcium cations have been shown to interact with gramicidin A channels at the mouth sites and block the conduction of monovalent cations across the cell membrane [22,33,38].

In conclusion, we demonstrate that the molecular state of gramicidin A is highly influenced by the concentration of calcium salt and the types of halide anion. Gramicidin A exists in two molecular states. In State 1 (concentrations ≤ 10 mM), the majority of gramicidin A forms a dimer. The addition of calcium salt converts the four conformers into a single species that forms a left-handed parallel double helical structure. In State 2, the addition of calcium salt induces a dissociation of dimers into monomers and a further denaturation of the structured monomers into the unstructured monomers. The abilities of dissociation and denaturation are highly dependent on the type of halide anion. Regarding antimicrobial activity, gramicidin A in the structured monomeric state showed the most effective antimicrobial activity. As the molecular state of gramicidin A is highly dependent on the dissociation ability of the halide anions, the dissociation rate can determine the molecular state and antimicrobial activity. Taken together, our study suggests that the molecular state may play a vital role in the antimicrobial activity.

## 4. Materials and Methods

### 4.1. Materials

Gramicidin A was purchased from Merck (Darmstadt, Germany). Spectrograde methanol and methyl-d3-alcohol were purchased from Sigma-Aldrich (St. Louis, MO, USA). Calcium chloride, calcium bromide and calcium iodide were purchased from Sigma-Aldrich (St. Louis, MO, USA). All chemicals were reagent grade and used without further purification.

### 4.2. Circular Dichroism (CD) Spectroscopy

Circular dichroism spectra were recorded using a J-815 CD spectrometer (JASCO International Co. Ltd., Tokyo, Japan) or a Chirascan-plus qCD spectrometer (Applied Photophysics, Surrey, UK). All measurements were performed in a quartz cell with a pathlength of 0.1 mm. The CD spectra of 100 μM gramicidin A in the presence of calcium salts with a designated concentration were collected from 200 to 250 nm with a 0.5 nm interval at 25 °C. The reported circular dichroism spectra were corrected with baseline using methanol containing the same concentration of salt and smoothed using a Savitsky–Golay function in Origin 6.0.

### 4.3. Mass Spectroscopy

Gramicidin A samples (100 µM each) were mixed with CaCl_2_, CaBr_2_ or CaI_2_ at ten different concentrations (0, 0.02, 0.07, 0.2, 2, 10, 40, 100, 200 and 300 mM) dissolved in either D_2_O or methanol. These samples were analyzed using MALDI-TOF mass spectrometry.

The matrix used for the analysis was α-cyano-4-OH cinnamic acid (CHCA). A 10 mg amount of CHCA was weighed and dissolved in 1.0 mL of 50% acetonitrile, 50% distilled H_2_O and 0.1% trifluoroacetic acid (TFA). The gramicidin A samples were then mixed with the CHCA matrix by adding 1 µL of the sample with 1 µL of the CHCA matrix. After mixing the sample and matrix well, 1 µL of the resulting mixtures were then spotted on to the MALDI plate for analysis. The MALDI spectra in the reflection mode were recorded with a Bruker New ultrafleXtreme^TM^ MALDI-TOF/TOF mass spectrometer from Bruker Daltonik, Bremen, Germany, at the Academia Sinica Institute of Chemistry Mass Spectrometry Center.

### 4.4. Nuclear Magnetic Resonance (NMR) Spectroscopy

We analyzed gramicidin A (100 μM) with CaCl_2_, CaBr_2_ or CaI_2_ (0.02, 0.2, 10 and 200 mM) dissolved in 90% d4-methanol/10% methanol using solution NMR. The NMR spectra were obtained using a Bruker DRX-500 NMR spectrometer equipped with a TXI z-gradient (1H, 13C, 15N) probe at 300 K (Bruker Biospin GmbH, Rheinstetten, Germany). In these spectra, the OH signal of methanol was suppressed using the presaturation method. All the NMR spectra were phased and baseline-corrected using the Topspin software (version 3.2.2; Bruker Biospin GmbH, Rheinstetten, Germany) and referenced to the chemical shift of methanol at 3.3 ppm.

### 4.5. Steady-State Fluorescence Measurements

All steady-state fluorescence measurements were performed with a Jasco FP-6500 fluorescence spectrometer (Tokyo, Japan) equipped with a water circulator and stirring accessory. The emission spectra of 100 μM gramicidin A in the presence of calcium halide salts at designed concentrations were recorded from 310 to 500 nm with an excitation wavelength of 290 nm. The bandwidths for excitation and emission were 3 and 5 nm, respectively. All measurements were performed in a quartz cell with a path length of 1 cm. The background spectra of the salts alone were taken first for later subtraction. The reported spectra were the averages obtained from at least three individual samples and three repeated measurements of each sample. All measurements were carried out at 25.0 ± 0.5 °C.

### 4.6. Bacterial Growth Assay

*Staphylococcus aureus* (*S. aureus*) (ATCC-25923) was kindly provided by Dr. J.W. Liou in Tzu Chi University. The *S. aureus* was grown in Luria–Bertani (LB) broth medium in a 250 mL flask at 37 °C overnight. This overnight *S. aureus* culture was then diluted to OD_600_ = 0.1 with LB medium. This *S. aureus* was then grown to lag phase (OD_600_ = 0.2), exponential phase (OD_600_ = 0.6) and late-exponential-to-stationary phase (OD_600_ = 1.5). At the designated growth phase, 5 mL of *S. aureus* culture was treated at 5% (*V*/*V*) with a stock solution containing gramicidin A (a final concentration of 5 μM) and CaCl_2_. The stock solution was prepared in the same way as used for the spectroscopic studies, in which a final concentration of 100 μM gramicidin A was dissolved in methanol containing 0, 0.2, 10, 100 and 400 mM of CaCl_2_. The *S. aureus* samples treated with or without gramicidin A/CaCl_2_ were incubated at 37 °C. The optical density at a wavelength of 600 nm, (OD_600_), was used to determine the growth curve of *S. aureus* using a microplate reader (FlexStation 3, MD) every 30 min.

## Figures and Tables

**Table 1:** Molar ratio of dimer/monomer of gramicidin A in the presence of CaCl_2_, CaBr_2_ and CaI_2_ at various concentrations.

Salt Concentration	Ratio (CaCl_2_)	Ratio (CaBr_2_)	Ratio (CaI_2_)
0.2 mM	6.0	2.0	0.5
10 mM	4.2	1.5	0.25
40 mM	2.0	0.5	0.22
100 mM	0.2	0.15	NA
400 mM	<0.1	<0.1	NA

## Supplementary Materials

Supplementary materials can be found at https://www.mdpi.com/1422-0067/21/17/6177/s1.
