# Supplementary material for: The Effect of Calcium and Halide Ions on the Gramicidin A Molecular State and Antimicrobial Activity
Source: Int J Mol Sci. 2020 Aug 27;21(17):6177. doi: 10.3390/ijms21176177 (PMC7503548; doi:10.3390/ijms21176177)
Supplement: Supplementary file 1 [file ijms-21-06177-s001.pdf]

Supplementary Information

The effect of calcium and halide ions on gramicidin A molecular state and antimicrobial activity

Katherine D. Carillo[1, 2, 3#], Chi-Jen Lo[4#], Der-Lii M. Tzou[3, 5#], Yi-Hung Lin[6], Shang-Ting Fang[7], Shu-Hsiang Huang[7], Yi-Cheng Chen[7*]

1. International Graduate Program, SCST, Academia Sinica, Nankang, Taipei 11529, Taiwan ROC
2. The Department of Applied Chemistry, National Chiao-Tung University, Hsinchu 30013, Taiwan, ROC
3. Institute of Chemistry, Academia Sinica, Nankang, Taipei 11529, Taiwan ROC
4. Metabolomics Core Laboratory, Healthy Aging Research Center, Chang Gung University, Taoyuan 333, Taiwan
5. Department of Applied Chemistry, National Chia-Yi University, Chia-Yi 60004, Taiwan, ROC
6. Department of Medicine, MacKay Medical College, New Taipei City 252, Taiwan
7. Experimental Facility Division, Industrial Application Group, National Synchrotron Radiation Research Center, Hsinchu Science Park, Hsinchu, 30076, Taiwan, R.O.C.

*Corresponding author: chen15@mmc.edu.tw

# Authors contribution equally

C: CaI_2

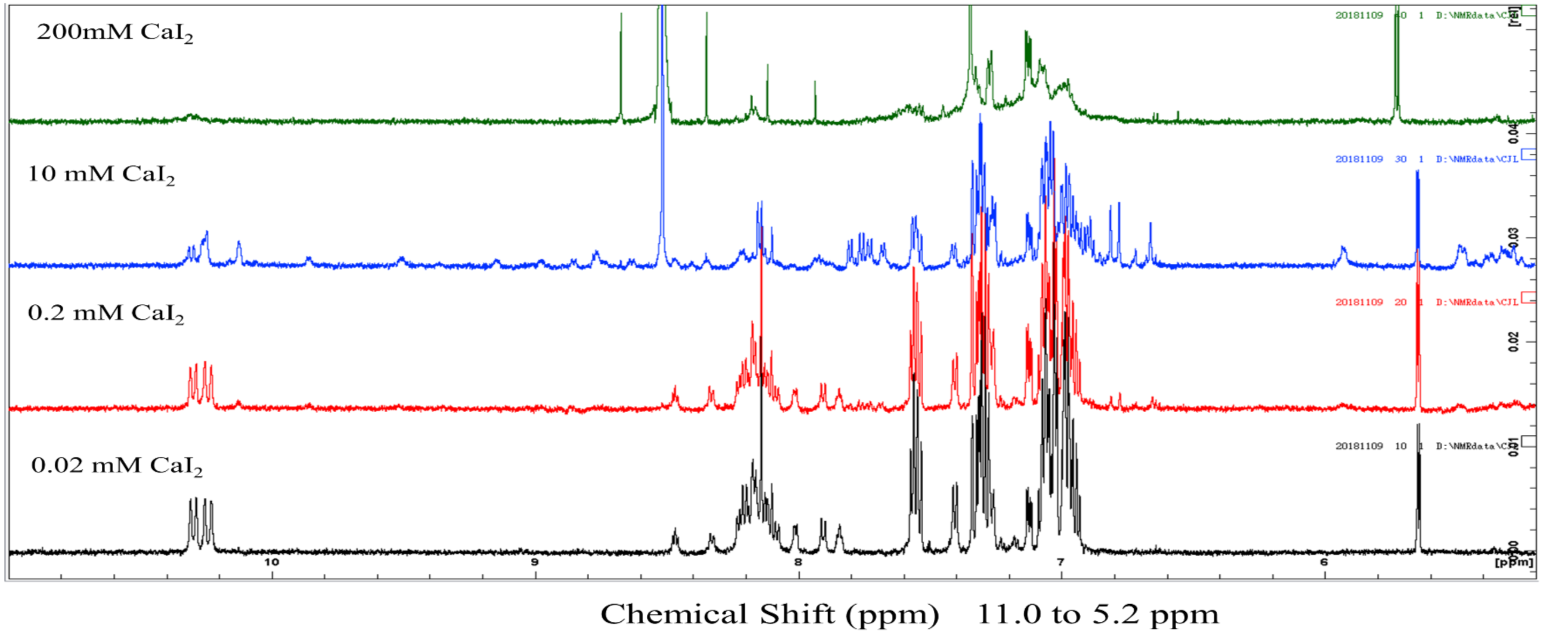

Figure S1. The amide region of NMR spectra of 100 μM gramicidin A in the presence of 0.02 mM, 0.2 mM, 10 mM and 200 mM CaI_2.

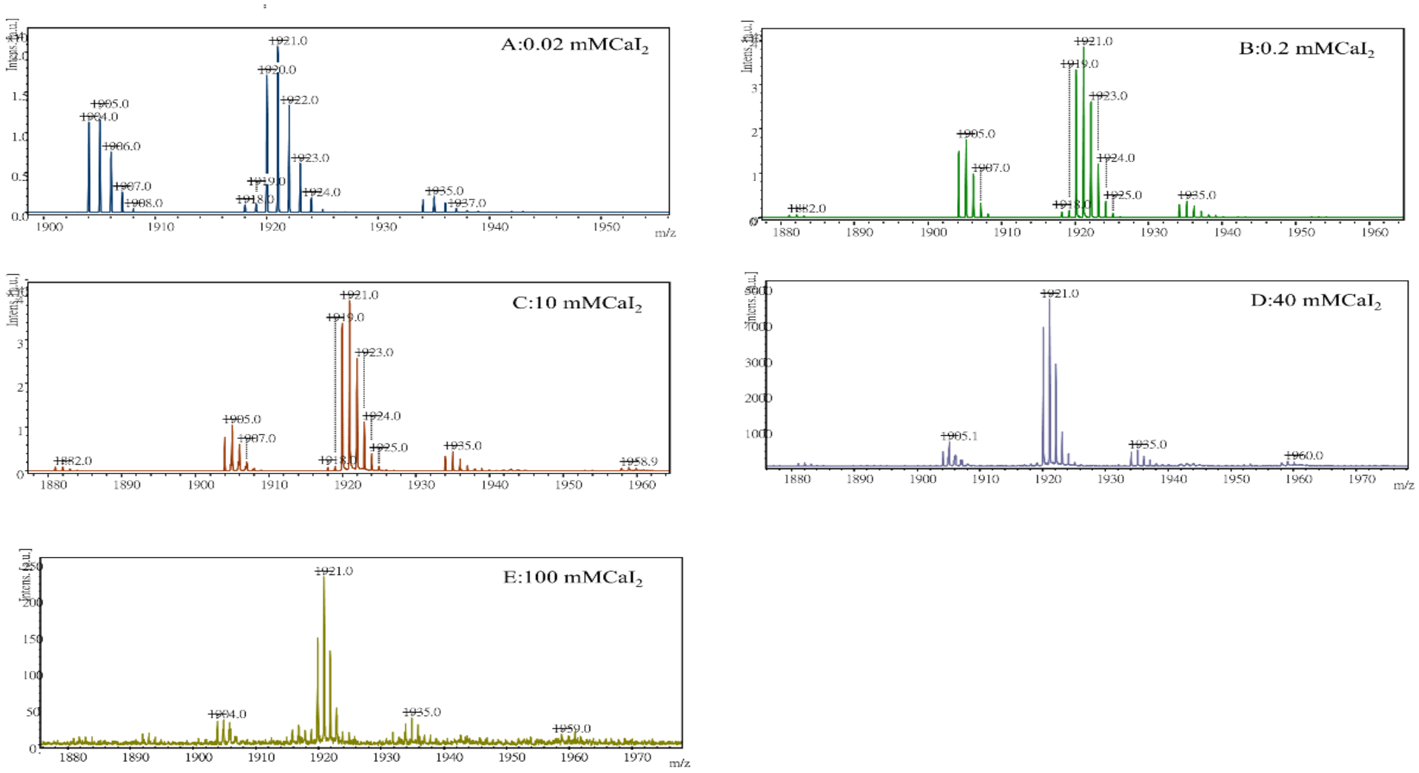

Figure S2. The mass spectra of gramicidin A in the presence of CaI_2. One hundred μM of gramicidin A was dissolved in methanol containing (A) 0.02 mM, (B) 0.2 mM, (C) 10 mM, (D) 40 mM and (E) 100 mM of CaI_2.

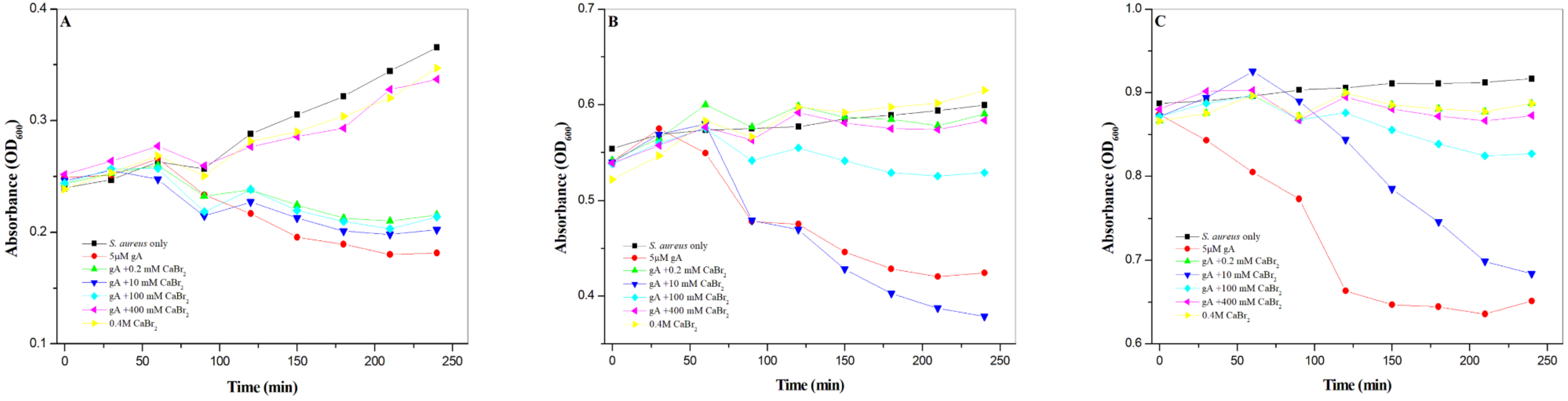

Figure S3. The antimicrobial activity of gramicidin A in the presence of $CaBr_2$ (A-C), respectively. In antimicrobial activity assay, stock solutions were prepared containing 100 μM of gramicidin A with 0, 0.2, 10, 100 and 400 mM of $CaBr_2$ in methanol. The stock solutions were then diluted into *S. aureus* culture with a final solution containing 5 μM gramicidin A. (A), lag phase. (B), exponential phase and (C), stationary phase, respectively.
